# Silica-Based Stimuli-Responsive Systems for Antitumor Drug Delivery and Controlled Release

**DOI:** 10.3390/pharmaceutics14010110

**Published:** 2022-01-04

**Authors:** Avelino Corma, Pablo Botella, Eva Rivero-Buceta

**Affiliations:** Instituto de Tecnología Química, Universitat Politècnica de València-Consejo Superior de Investigaciones Científica, 46022 Valencia, Spain; evribu@upvnet.upv.es

**Keywords:** silica nanoparticles, drug delivery, stimuli-responsive, controlled release, cancer therapy, camptothecin, docetaxel, doxorubicin

## Abstract

The administration of cytotoxic drugs in classical chemotherapy is frequently limited by water solubility, low plasmatic stability, and a myriad of secondary effects associated with their diffusion to healthy tissue. In this sense, novel pharmaceutical forms able to deliver selectively these drugs to the malign cells, and imposing a space-time precise control of their discharge, are needed. In the last two decades, silica nanoparticles have been proposed as safe vehicles for antitumor molecules due to their stability in physiological medium, high surface area and easy functionalization, and good biocompatibility. In this review, we focus on silica-based nanomedicines provided with specific mechanisms for intracellular drug release. According to silica nature (amorphous, mesostructured, and hybrids) nanocarriers responding to a variety of stimuli endogenously (e.g., pH, redox potential, and enzyme activity) or exogenously (e.g., magnetic field, light, temperature, and ultrasound) are proposed. Furthermore, the incorporation of targeting molecules (e.g., monoclonal antibodies) that interact with specific cell membrane receptors allows a selective delivery to cancer cells to be carried out. Eventually, we present some remarks on the most important formulations in the pipeline for clinical approval, and we discuss the most difficult tasks to tackle in the near future, in order to extend the use of these nanomedicines to real patients.

## 1. Introduction

In recent years, nanoparticles have emerged as key players in modern medicine, with applications ranging from contrast agents in medical imaging to gene delivery carriers in individual cells. An increasing number of nanotherapeutic drugs have already been commercialized or reached the clinical stage [1]. In the case of oncologic applications, and compared to simple molecule therapies, currently most FDA-approved nanoparticle-based drug delivery systems (DDSs) are being designed for the re-formulation of combinations of chemotherapeutic drugs, looking for enhanced pharmacokinetics (PK), biocompatibility, tumor-targeting, and stability, while simultaneously minimizing systemic toxicity and overcoming drug resistance [2]. Furthermore, the possibility of introducing tracking moieties to promote medical imaging leads to the development of efficient theranostic systems, which are able to carry out diagnostic and therapy in one go [3].

In this context, the use of silica nanoparticles (SNPs), and especially of mesoporous silica nanoparticles (MSNs), in drug delivery was formerly based on their physical and textural properties, with empty mesoporous channels to absorb relatively large amounts of bioactive molecules. Different groups have systematically studied the influence of pore diameter, pore structure, surface area, and pore volume on drug loading and release rate [4,5]. It has been shown that the decrease in pore diameter leads to a decrease in drug-loading quantity and release rate. At the same time, the pore structure type in terms of pore connectivity may condition the diffusion process and, in this sense, a one-dimensional pore structure with cage-like pores is the most promising pore geometry for providing high drug-loading amount and slow drug release. Additionally, both pore volume and surface area favor the incorporation of drug molecules within the mesoporous structure.

The incorporation of drugs in SNPs can take place through non-covalent interactions, such as hydrogen bonding, physical adsorption, electrostatic interaction, and π–π stacking [6,7]. Unfortunately, in most cases, these kinds of interactions are very weak, and some or total premature release of the cargo may occur before reaching the destination. The premature release problem not only limits the use of a DDS for effective therapy, but also plays a major challenge on possible side effects that can be related to the activity of the active principle outside the targeted cells or tissue. In this sense, surface functionalization of SNPs with appropriate organic groups allows for the incorporation of the therapeutic molecules by more stable interacting forces, such as ionic bond and covalent bond. These functionalized mesoporous SNPs are highly stable DDSs, able to deliver the drug with no leakage before reaching the designated site of cells or tissue. 

Furthermore, silica surface can be engineered with specific organic moieties for the development of stimuli-responsive systems (SRSs), that is, delivery nanostructures that release their cargo under the action of a specific stimulus [8]. When used as drug carriers, these stimuli-responsive nanoparticles are good candidates for strong therapeutic activity with no toxicity effects. A wide range of different SRSs can be classified as endogenous or exogenous, depending on the nature of the stimulus (internal or external) used to release the therapeutic agent at the specific site without premature release. However, these “smart” systems can be tailored to respond selectively to (i) internal stimuli such as pH, redox, enzyme, or temperature; and (ii) external stimuli such as magnetic field, light, and ultrasound [9,10,11]. It is important to note that charge release, in both cases, occurs via a different pathway. While SRSs that respond to internal stimuli take advantage of the differences between cancerous and normal tissue environments, SRSs that are sensitive to external stimuli modify their characteristics or properties in the presence of a physical event. One of the main advantages of these “smart systems” is that, by controlling the release of the drug in a specific area of the tissue, they allow, on the one hand, side effects to be minimized and, on the other hand, efficacy of the treatment to be improved [10].

At this point, selective cancer therapy needs to develop methodologies to target malignant cells and minimize the impact on healthy tissue. For this purpose, different components have been used as targeting moieties, as small molecules, peptide sequences, polysaccharides, aptamers, and antibodies. Actually, recent studies have been focused on cancer therapy with targeting molecules, such as aptamers and monoclonal antibodies [12,13]. The use of monoclonal antibodies for tumor targeting of drug delivery platforms is an important tool for clinical applications, due to their high affinity, specificity, and versatility. The term ‘affinity’ refers to the strength of the interaction between a single region of the monoclonal antibody and a single antigen. In this strategy, antibodies bind specifically to the corresponding antigens overexpressed on the surface on cancer cells, which can lead to selective drug accumulation at the tumor site [14]. The main benefit of this strategy is the reduction in adverse effects by selective interactions between antibody and cell-surface receptors [15].

We here summarize the most advanced and recent achievements in the design, synthesis, and development of silica-based SRSs for cancer therapy, as well as the influence of targeting on the final efficacy. Moreover, we have also introduced a brief description of the most important formulations that are currently in the pipeline for clinical testing. 

## 2. Stimuli-Responsive Systems Based in Endogenous Activity

These nanodevices can be tailored by introducing breakable bonds or gatekeepers into the nanoparticle structure as pore blockers, which can degrade in response to an internal feature of the organism, including pH, enzymes, redox environment, and temperature. Some of the most significant proposed endogenous or internal stimulus-response systems are presented in Table 1.

### 2.1. pH-Responsive Systems

The pH difference between normal and tumor tissues is well-known. Tumor tissues have a slightly acidic pH in both endosomes and lysosomes compared to normal tissues, which can be used to trigger drug release from pH-sensitive nanoplatforms [16]. Therefore, drug release only occurs when it comes into contact with the tumor microenvironment. In general, these SRSs contain protonable chemical groups (i.e., amino, imidazolyl, and carboxyl groups), that are sensitive to acidic pH. These groups are easily protonated at acid pH, causing destabilization of the structure and, therefore, the release of the drug [17,18]. 

**Figure 1:**
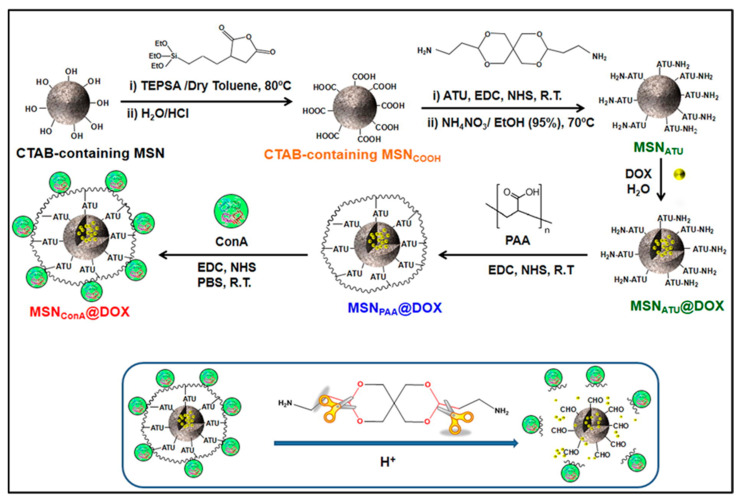
Schematic illustration of the synthesis steps involved in preparing dual pH and light sensitive mechanized SNPs. (Reprinted with permission from reference [19]. Copyright © 2022 Acta Materialia Inc. Published by Elsevier Ltd., Amsterdam, The Netherlands).

In a recently published study, a pH-sensitive system for drug delivery was obtained by conjugating a biocompatible hydrophilic polymer of polyacrylic acid (PAA) used as a pore-blocking agent and MSNs via an acid-cleavable acetal linker [19]. For this purpose, MSNs were grafted with the acid-sensitive linker 3,9-Bis(3-aminopropyl)-2,4,8,10-tetraoxaspiro [5.5] (ATU) to load the DOX chemotherapeutic agent and to seal the pores with the PAA polymer (Figure 1). Next, the lectin concanavalin A (ConA), which acts as a targeting molecule, and is noted for its ability to recognize and bind to cell surface glycans selectively, is grafted onto the PAA polymer.

In vitro cytotoxic assays of MSN_ConA_@DOX against HOS human osteosarcoma cell line, which overexpress sialic acid, showed more potent cytotoxicity than nanoparticles without ConA. Here, no significant differences were observed in the MC3T3-E1 preosteoblastic cell line (which does not overexpress sialic acid). This may be due to the fact that nanoparticles conjugated with ConA on the surface can interact with sialic acid, which is overexpressed in HOS cells compared to MC3T3-E1 cells, favoring the internalization of nanoparticles selectively into HOS cells.

**Table 1:** Types of MSN-based internal stimuli-responsive systems for drug delivery.

Stimulus	Drug Loading	Release System	Release Mechanism	Ref.
pH	Doxorubicin	MSNs grafted with the pH sensitive linker ATU and coated with the acid degradable polymer PAA	Acid-cleavable acetal (ATU) linker	[19]
	Doxorubicin and pheophorbide a	Hollow MSNs decorated with chitosan as a capping layer and GPTMS as crosslinking and attaching agent	At acidic pH, the CS/GPTMS layer swells, leaving the pores free.	[20]
	Doxorubicin	MSNs conjugated with supramolecular switches forming by hydrazone bond, azobenzene and α-cyclodextrin	Hydrolyzation of acid-sensitive hydrazine bonds	[21]
	Sulforhodamine B	MSNs with functionalized pore walls and grafted with a pH-responsive cross-linked polymer pDAEM	Protonation/deprotonation of tertiary amines of polymer	[22]
Redox	Camptothecin (CPT)	Silica hybrid nanoparticles conjugated with pyridine-2-yldisulfanyl)alkyl carbonate derivatives of CPT	Disulfide reduction, intra-molecular cyclization, and dissociation of nanoparticles	[23,24]
	Pyrene	Spherical PLGA nanoparticles containing hydrophobic molecules covered by a thin layer of a redox-responsive amorphous organosilica shell	Disulfide bridge reduction and pore opening	[25]
	Hydroxycamptothecin (HCPT)	Disulfide-doped organosilica-micellar hybrid nanoparticles	Two stage rocket-mimetic redox responsive mechanism. First, detachment of disulfide-bond of PEG and second, degradation of disulfide-doped silsesquioxane framework	[26]
	Ribonuclease A (RNase A)	Diselenide-bridged mesoporous SNPs	Degradation of diselenide bridge in oxidative and reduction conditions	[27]
Enzyme	Doxorubicin	Hollow MSNs grafted with chitosan as a gatekeeper by an azo linkage	Degradation of azo bonds	[28]
	Doxorubicin	Hybrid nanospheres composed of an organic core (liposome) and an inorganic shell formed by ester fragments bonded covalently to silica units	Ester bond hydrolysis	[29]
	Camptothecin	Amorphous SNPs decorated with CPT	Ester bond hydrolysis	[30]
	Docetaxel (DTX)	MSNs conjugated with DTX and a PSMA antibody	Ester bond hydrolysis	[31]
Temperature	Doxorubicin hydrochloride	Magnetic MSNs coated with polymer poly(N-isopropylacrylamide-co-acrylamide) as a gate-keeper	Conformational change in thermoresponsive polymer P(NIPAM-co-MAA)	[32]
	Rhodamine 6G	Solid core mesoporous shells and nonporous solid corer SNPs grafted with poly(N-isopropylacrylami-de) brushes	Conformational change in thermoresponsive polymer PNIPAM	[33]
	Doxorubicin	Hollow MSNs coated with poly(N-isopropylacrylamide) modified with metha acrylamide (Mam) and with Fe_3_O_4_ nanoparticles embedded in the polymer shell	Conformational change in thermoresponsive polymer P(NIPAM-Mam)	[34]

Biodegradable hollow mesoporous silica nanoparticles (HMSNs) capped with chitosan (CS) were prepared by a selective etching method to overcome the problem of nanosilica retention issue in the body [20]. Once the HMSNs were prepared, a chemotherapy agent (Doxorubicin, DOX) and a photosensitizer (Pheophorbide a, PA) were loaded in the hollow core and mesopores of the HMSNs. Then, the chitosan was cross-linked with glycidoxypropyl-trimethoxy-silane (GPTMS) and self-assembled on the surface of HMSNs via siloxy bonds. Taking into account that the folic acid (FA) receptor alpha (FR) is overexpressed on the surface of different tumors, FA was incorporated into the nanoparticles as a target molecule, thus achieving a more selective drug delivery, reducing side effects (HMSN-GM-CS-FA@DOX/PA). Drug release was studied at different pH values (7.4, 6.8, and 5.0), finding that while at pH 7.4, there is no evident release, and at pH 6.8, there is a slight release of PA and DOX, which increases drastically at pH 5. In vitro cytotoxicity assays on KB human buccal epidermal carcinoma cells showed a significant increase in the toxicity effect of nanoparticles under irradiation, confirming that the presence of different therapeutic agents in the nanoparticles simultaneously causes a synergistic effect that result in enhanced tumor inhibition. In addition, the presence of folic acid as a directing molecule favors the accumulation of the particles at the tumor site and the selective interaction with FR receptors on the cell membrane, allowing the nanoparticles to be internalized from the cell surface into the intracellular compartment by receptor-mediated endocytosis. Furthermore, in vivo assays in female BALB/c nude mice confirmed that the combination of different agents in a single formulation is a strategy that achieves more significant tumor growth inhibition.

Other dual pH and light sensitive mechanized silica nanoparticles (MSNPs) were able to deliver chemotherapeutic drugs in response to single or multiple stimuli simultaneously [21]. This nanoplatform was based on MSNs functionalized with hydrazinylmethylene azobenzene (HMAB) through hydrazone bonds as pH-sensitive linkers. After loading the cargo, α-CD macrocycles were incorporated into the functional stalks, which act as caps and block the pores to prevent cargo leakage. In this case, drug release can be accomplished by a change in pH or UV irradiation at 365 nm. In both cases, the macrocycles are detached by different mechanisms, resulting in the release of DOX molecules. The drug release was compared at pH 7.4, 6.0, and pH 5.0, concluding that in an acidic medium, the amount of DOX released increases up to 57.5 and 75.2%, respectively, caused by the collapse of the labile hydrazone bonds at acidic pH. Although, in vitro cytotoxic assays against MCF-7 human breast cancer cells suggested high anticancer activity, both for DOX-loaded MSNPs with or without irradiation. Furthermore, cell internalization studies showed that DOX release occurred slowly into the cytoplasm and then spread to the nucleus, causing cell death. In the presence of UV irradiation, DOX release was observed much more efficiently.

**Figure 2:**
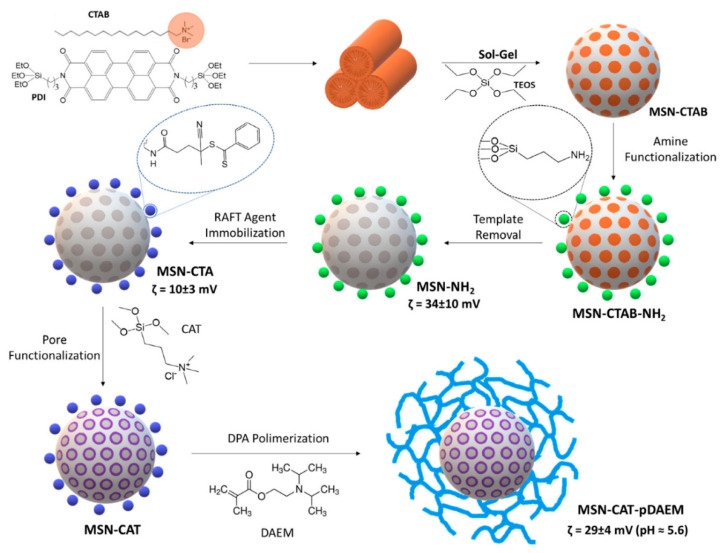
Schematic illustration of the synthesis steps involved in preparing pH SNPs. (Reprinted with permission from reference [22]. Copyright © 2022, MDPI, Basel, Switzerland).

A new hybrid nanocarrier was developed to control charge release as a function of the pH of the medium (Figure 2). This system is composed of a mesoporous silica core and a pH-sensitive polymer shell, which functions as a switch that allows it to retain or release the charge [22]. On the fluorescent MSNs, which incorporate perylenediimide derivative (PDI) for optical tracking of the nanoparticles, functionalized with amino groups, a chain transfer agent (MSN-CTA) was immobilized and was responsible for polymerizing the shell by RAFT polymerization approach. Before the polymerization of the nanoparticle surface (MSN-CAT-pDAEM), the pores were functionalized with a cationic molecule, *N*-trimethoxysilylpropyl-*N*,*N*,*N*-trimethylammonium chloride (CAT) (MSN-CAT). 

In addition, different tests were performed at different pH values to determine the effect of the functionalization of the pore walls and the presence of the polymer coating on the release of sulforhodamine B (SRB). In a first study performed at pH 7.5, it was found that nanoparticles with functionalized pore walls (MSN-CAT) showed a much slower SRB release, approximately 10 times slower, than non-functionalized particles. Conversely, the release profile of these systems was characterized at acidic and basic pH values, revealing that the interaction between the SRB (anionic molecule) with the polymeric shell is much stronger at low pH (cationic chains) due to electrostatic attraction, resulting in efficient charge retention in the polymeric network. When the pH value increases, the electrostatic interaction disappears, and the collapse of the polymer shell squeezes the charge out. The combination of these two properties results in an ON:OFF system for controlled charge release. 

### 2.2. Redox-Responsive Systems

The significant difference in glutathione (GSH) concentration at intracellular and extracellular levels, and its higher concentration in tumor tissues compared to normal tissues, provide a potentially feasible strategy for targeted therapy [35]. The tumor-reducing environment is used as a strategy to design such DDSs. These kinds of systems incorporate redox-sensitive ligands in their structure that enables the control of cargo release in tumor cells. The redox-sensitive bonds commonly used are the disulfide bridge, the diselenide bond, and the thioether bond. In this case, hydrophobic molecules can be loaded on the inner core, pores, or covalently attached to nanoparticles’ surface, which allows the designing of a wide variety of responsive systems [36].

**Figure 3:**
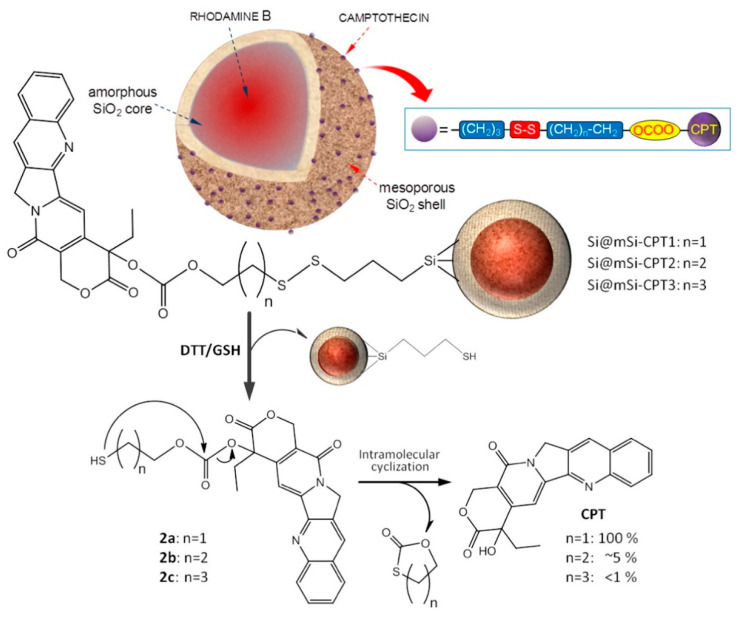
Schematic representation of the redox-sensitive release system based on hybrid mesoporous SNPs surface functionalized with (pyridin-2-yldisulfanyl)alkyl carbonate derivatives of CPT. (Reprinted with permission from reference [24]. Copyright © 2022 Elsevier B.V., Amsterdam, The Netherlands).

More recently, our group has developed different redox-responsive nanoplatforms through a new synthetic strategy, based on the direct conjugation of (pyridin-2-yldisulfanyl)alkyl carbonate derivatives of CPT with cleavable disulfide linkers to hybrid SNPs containing a non-porous core and a mesoporous shell [23,24]. This research aims to determine the release mechanism and properties of the prepared materials. In addition, with the incorporation of a fluorophore into the nanoparticles, it has been possible to determine by confocal microscopy in HeLa cells that the entry of the nanoparticles into the cells occurs by endocytosis. To complete this work, we determined how the incorporation of CPT prodrugs with different number of carbons in the side chain affects the properties of hybrid porous SNPs [24]. The most important conclusion is that the number of side-chains determines the properties of the resulting materials, including hydrophobic properties, and the type of released products and cytotoxic activity. When the number of side-chain carbons increases, the negative surface charge decreases, favoring particle aggregation and cell internalization. Although complete reduction in the disulfide bridge occurs in all cases inside the cell, the release of the free drug by intramolecular cyclization only occurs in homologues with n = 1. However, in homologues with n = 2 and 3, the carbon bond is slowly hydrolyzed, leading to the release of small amounts of CPT (Figure 3). 

As a different strategy, we decided to prepare hybrid nanoparticles based on a PLGA nanoparticle core and an amorphous organosilica shell with redox-sensitive molecular gates [25]. These spherical PLGA nanoparticles have the characteristic of containing hydrophobic molecules making their storage possible. Additionally, they were coated by a thin layer of silica, which incorporates disulfide bonds as molecular gates that can be selectively cleaved by reducing agents and includes features of silica-based materials, such as chemical inertness, easy regulation of the coating process, porosity, and biocompatibility. This system provides greater control and slower release of the encapsulated molecules. In addition, they are reasonably stable in a physiological environment and potentially sensitive to redox mechanisms.

A new type of gradient redox-responsive and two-stage rocket mimic drug delivery system was developed by incorporating bifunctional polymers on organosilica hybrid nanoparticles to improve tumor accumulation and therapeutic efficacy [26]. For this purpose, disulfide-doped organosilica hybrid nanoparticle (DOSN-COOH-SH) with doubly functionalized carboxyl/thiol groups was synthesized via hydrolysis/condensation on poly(ε-caprolactone)-bpoly(acrylic acid) (PCL-b-PAA) micellar template. Next, the PEG chains functionalized with thiol groups were covalently conjugated on the surface by an oxidation reaction (DOSN-COOH-SS-PEG). In the final step, the positively charged PEI molecules were covalently bonded by amidation (DOSN-PEI-SS-PEG). Considering the structure of this nanoplatform, this model relies on the difference in glutathione levels between the tumor environment and the tumor cells to carry out the disintegration of the system in two stages. While in the first stage, the PEG chains are detached (GSH concentration 2–20 µM), in the second stage, the disulfide-doped silsesquioxane structures are degraded (glutathione concentration 2–10 mM). 

On the other hand, the nanoparticles were loaded with hydroxycamptothecin (HCPT) for biological validation. In vitro efficacy assays showed that the inhibition activity of HCPT@DOSN-PEI-SS-PEG was superior to that of the naked drug against SMMC-7221 human cancer cells. Still, in normal NIH/3T3 rat fibroblasts, the inhibition obtained is lower than that of the free drug. Furthermore, confocal microscopy experiments confirmed that these nanoparticles are internalized through the lipid raft-mediated endocytosis pathway. Finally, an in vivo study in SMMC-7221 nude mice indicated that HCPT@DOSN-PEI-SS-PEG nanoparticles enhance therapeutic efficacy and exhibit an excellent biocompatible profile and low toxicity. 

**Figure 4:**
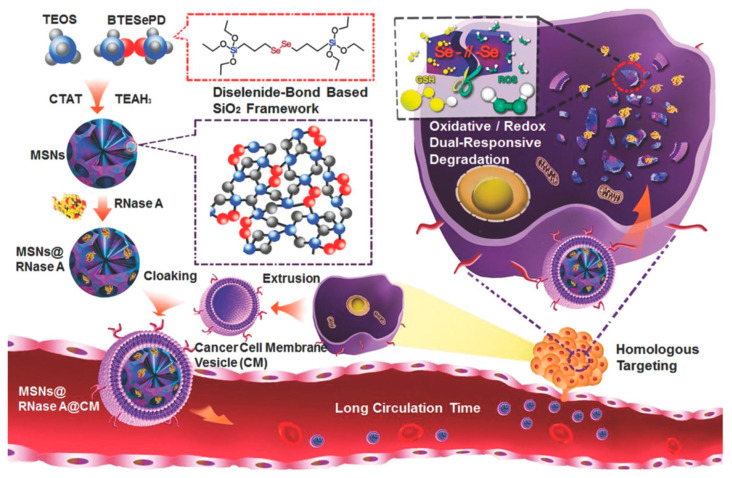
Schematic representations of the synthesis of diselenide-bond-bridged MSN and its mechanism of action. (Reprinted with permission from reference [27]. Copyright © 2022 WILEY-VCH Verlag GmbH & Co. KGaA, Weinheim, Germany).

In another study, a dual redox/oxidative response nanoplatform was prepared from diselenide-bridged MSN structures, which can cleave in the presence of redox or oxidative conditions and thus release the charge at the specific site [27]. To evaluate the matrix’s loading capacity and degradation-controlled release behavior, in this prototype (Figure 4), the cytotoxic enzyme RNase A was incorporated into the nanoparticle pores via electrostatic interactions (MSN@RNase A). Furthermore, RNase A-loaded MSNs were coated with cancer cell membrane (CM) to obtain a biomimetic nanoplatform (MSN@RNase A@CM). Coating the nanoparticles with the CM is a very efficient strategy, since this biomimetic nanoplatform of cancer cells membranes exhibits homologous targeting and immune properties inherited from the cancer cells of origin, which could increase blood circulation time, enhance accumulation in the tumor, and sustained release of bioactive protein.

These nanoparticles showed excellent cell internalization ability in HeLa human cervical carcinoma cells, confirming by intracellular colocalization that the cancer cell membrane must cause the enhanced internalization. Furthermore, cytotoxicity studies in HeLa cells showed that nanoparticles loaded with RNase A and CM provided slightly higher anticancer activity compared to nanoparticles that did not incorporate selenium in their structure. This could be explained by the rapid degradation of diselenide bonds due to the oxidative/reductive environment of cancer cells leading to the release of the cargo. In vivo assays in female nude mice bearing orthotopic HeLa tumors showed that treatment with RNase A-loaded nanoparticles with/without CM reduced tumor volumes and tumor weight due to accumulation of the nanoparticles in the tumor caused by CM camouflage. However, no significant variations in biochemical parameters and major organs were observed.

### 2.3. Enzyme-Responsive Systems

Enzyme-responsive nanomaterials take advantage of the overexpression and deregulation of numerous enzymes caused by diseases such as cancer and other pathologies. These act as release triggers through enzymatic digestion of the nanocarrier or linker moiety [37,38]. In this type of nanoplatform, the therapeutic molecule is conjugated to the nanoparticles through covalent cleavage bonding or physical encapsulation [39]. The high number of enzymes overexpressed in the tumor environment is responsible for degrading the structures of the drug delivery systems or causing the cleavage of the bonds so that the drug is released at the specific site.

In a unique approach, doxorubicin was encapsulated in a hollow mesoporous silica sphere (HMSS) grafted with chitosan (CS) to achieve an enzyme-responsive colon-specific drug delivery system [28]. Next, HMSS nanoparticles were covalently conjugated by amidation to incorporate azo bonds on the nanoparticle surface (HMSS-N=N-COOH), which can be cleaved by the enzyme at colon sites. Subsequently, CS was incorporated by conjugation with HMSS-N=N-COOH via an amide bond (HMSS-N=N-CS). This biopolymer acted as a gatekeeper, thus preventing premature drug release. In addition, protein adsorption assays to evaluate the effect derived from the presence of CS on the surface of nanoparticles using bovine serum albumin (BSA) showed higher stability, biocompatibility, and lower protein adsorption on the surface of HMSS-N=N-CS. In this case, DOX was encapsulated in the cavities of the nanoparticles. While the obtained material was very stable under physiological conditions, under acidic conditions, the CS swelled rapidly due to protonation, leading to pore opening, and inducing DOX release. In vitro efficacy assays against Caco-2 human colorectal adenocarcinoma cells showed that intracellular release of the cargo induced cell death with half-maximal inhibitory concentration (IC_50_) values similar to those observed with the naked drug when HMSS-N=N-CS/DOX was pre-incubated with concentrated colon enzymes. This effect could probably be due to the colon enzymes degrading the azo bonds, which would trigger the detachment of the conjugated CS on the HMSS surface, accelerating the rapid release of DOX. Furthermore, HMSS and HMSS-N=N-CS showed good biocompatibility after oral administration for one week at an administration dose of 100 mg/kg, without marked histopathological changes or hyperthermia and with a slight increase in body weight.

**Figure 5:**
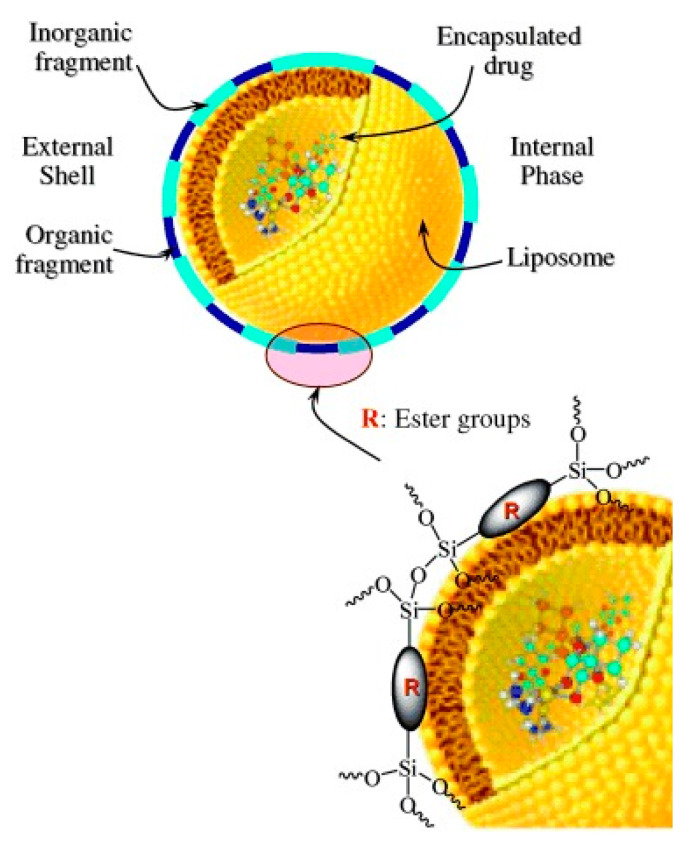
Schematic illustration of enzyme-sensitive organic–inorganic nanospheres. (Reprinted with permission from reference [29]. Copyright © 2022 WILEY-VCH Verlag GmbH & Co. KGaA, Weinheim, Germany).

As a different model, enzyme-sensitive organic–inorganic nanospheres were prepared in two steps, in which DOX molecules were directly grafted via ester linkages [29]. First, an inner liposomal organic phase is prepared in which doxorubicin molecules are encapsulated. Subsequently, an inorganic layer is incorporated around the liposomes, which is formed by bridging silsesquioxane esters (BTEPAA) covalently bonded to silica units (Figure 5). This material is stable at blood pH value due to the ester groups of the organic shell, and the presence of esterase enzymes favors its hydrolysis by saponification and the release of DOX. In vitro assays of these nanospheres against the human glioma cell line 42-MGBA showed that the nanoparticles entered the cells to release the cargo, and the induced cell death is similar to that observed for the naked drug. 

In recent years, in our group, we have been working on the design and development of different systems based on the incorporation of different molecules with therapeutic effect on the surface of a variety of nanoparticles through covalent bonding [30,31]. The synthetic steps for obtaining nanoparticles with CPT started with the esterification of *t*-Boc-glycine with CPT at the 20-OH position to give 20-O-trifluoroglycinylcamptothecin following a protocol described in the literature [40]. This prodrug was then conjugated to acid groups on the surface of amorphous SNPs by nucleophilic substitution (SNP-CPT) [30]. The SNP-CPT is a robust system that exhibited a longer plasma lifetime, with less than 10% of the total CPT released, which is most likely the result of lower enzymatic activity over these nanoparticles. However, the nanoparticles were labelled with Cy5.5 (SNP-CPT-Cy5.5) to establish the mechanism of cell internalization and cytotoxic activity. These in vitro efficacy assays against cervix (HeLa), primary glioblastoma (U87-MG), and colorectal (HCT-116 and HT-29) tumor cell lines showed internalization of the nanoparticles via endocytosis and cytotoxicity almost similar to that of free CPT. Moreover, to complete the biological validation, in vivo biodistribution and tolerability assays were performed in hsd:athymic nude-Foxn1 mice. The results demonstrated the effect of SNP-CPT on the tumor, causing the reduction of systemic toxicity associated with CPT administration and the suppression of tumor growth. However, it was observed that these nanoparticles accumulated significantly in organs of the reticuloendothelial system. 

### 2.4. Thermo-Responsive Systems

Thermosensitive nanocarriers are nanoparticles that can retain the therapeutic payload at human body temperature (37 °C). With an increase in temperature, taking advantage of the higher temperature of tumor cells than normal cells, drug release occurs [41]. These systems contain at least one component in their structure that has its properties altered with temperature, which undergoes a conformational change that compromises the integrity of the nanostructure and triggers the release of the payload. Generally, thermosensitive polymers are used to achieve this goal. Therefore, thermosensitive polymers are often used in the construction or design of these systems. These polymers exhibit a reversible phase transition that occurs in response to a change in temperature [42]. Typically, the polymers are designed so that their phase transition, referred as lower critical solution temperature (LCST), occurs at about 37 °C. Furthermore, the change in state occurs when the temperature is above the LCST [43].

In this context, a thermosensitive drug delivery system based on magnetic mesoporous silica nanoparticles (MMSNs) can be prepared. First, the surface of MMSN nanoparticles was functionalized with propyl methacrylate (MPS) [32]. Then, (N-iso-propylacrylamide-*co*-methacrylic acid) (P(NIPAM)-*co*-MAA) thermosensitized MMSNs by precipitation polymerization of N-isopropylacrylamide (NIPAM) and methacrylic acid (MAA) on methacrylate groups. While at 37 °C (<LCST), the polymer is hydrophilic and possesses an extended random coil conformation, and probably blocks the pores, at temperatures of 50 °C or higher (>LCST), it is hydrophobic, and the structure collapses, opening the pores and allowing drug release by diffusion. At 50 °C and pH 5, DOX release from the nanoparticles (DOX-MMSN@P(NIPAM-co-MAA)) was faster than at 37 °C (pH 5 or 7.4) due to temperature-induced structural alterations in the NIPAM conformation and the acidic medium simulating the cancer cell environment. In vitro cytotoxicity studies performed by MTT assay on HeLa human cervical carcinoma cells showed that DOX-MMSN@P(NIPAM-*co*-MAA) nanoparticles have an antitumor effect enhanced by the treatment with a magnetic field, confirming a synergistic effect of chemotherapy and magnetic hyperthermia.

Another recently described example also uses the temperature-sensitive polymer PNIPAM to develop hybrid nanoparticles that can be used as reservoirs for molecules [33]. SNPs with solid cores (porous (SCMS) and non-porous (SC)), with a diameter of about 380 nm, and porous shells conjugated with thermosensitive PNIPAM brushes were synthesized using a two-step method. First, the nanoparticles were functionalized with amine groups and then conjugated with then α-bromoisobutyryl bromide (BIBB). Next, after template extraction with CTAB, PNIPAM brushes were grafted onto the nanoparticles (SCMS-PNIPAM and SC-PNIPAM) at different times to determine the influence of time on the polymerization process. Then, these nanoparticles were loaded with rhodamine 6G (Rh6G) by immersion in an aqueous solution. Thus, it could be observed that the SCMS-PNIPAM nanoparticles adsorbed the highest amount of rhodamine 6G inside the mesopores, even at 50 °C. Moreover, these nanoparticles showed that they could modulate the charge release by varying the methanol content. The amount of methanol used can be crucial to cause a transition change in the polymer and, thus, control the charge diffusion through the pores. While high or low amounts of methanol cause the polymer to swell, preventing it from releasing charge, a mixture of methanol and water aggregates the PNIPAM chains, causing the pores to open and effectively increasing the amount of lead released

**Figure 6:**
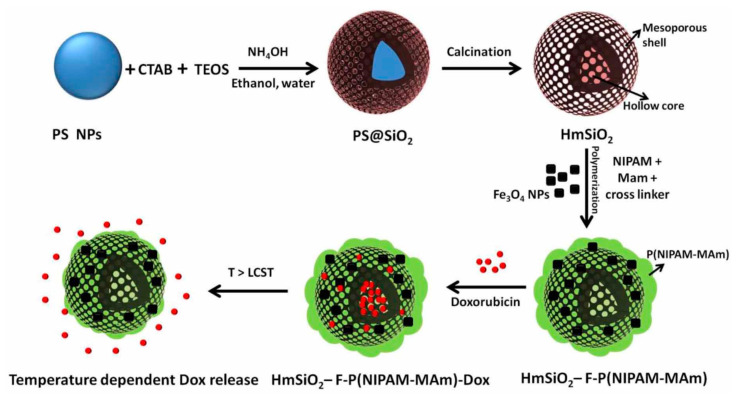
Schematic representation of the synthesis of hybrid hollow MSNs (HmSiO_2_) coated with a shell of P(NIPAM-MAm) with embedded Fe_3_O_4_ NPs. (Reprinted with permission from reference [34]. Copyright © 2022 IOP Publishing, Ltd., Bristol, UK).

In another model based on thermosensitive MSNs, hollow MSNs (HmSiO_2_) were coated with P(NIPAM-MAm) to generate a shell on HmSiO_2_ with embedded Fe_3_O_4_ NPs [34]. For this purpose (Figure 6), the surface of the HmSiO_2_ nanoparticles was functionalized with propyl methacrylate (MPS), and subsequently the polymeric shell was generated by covalent interaction between HmSiO_2_ nanoparticles and P(NIPAM-MAm) polymer. In the final stage of the synthesis, the iron nanoparticles were modified with oleic acid and embedded into the polymeric shell by hydrophobic interaction between the hydrophobic parts of the polymer and oleic acid (HmSiO_2_-F-P(NIPAM-Mam). Then, DOX was encapsulated in nanoparticles (HmSiO_2_-F-P(NIPAM-MAm)-DOX) by electrostatic interactions between the drug and HmSiO_2_. At a temperature above LCST (40 °C) or pH 5, the encapsulated DOX was rapidly released from these nanocarriers; although, at pH 7.4, the release rate was slower below LCST. In vitro cytotoxicity assays against HeLa tumor cells and normal cells confirmed that these nanoparticles showed superior cytotoxicity against tumor cells, probably because the slightly acidic value of these cells favors a rapid release of DOX. Although the system showed lower cytotoxicity against HeLa cells than free DOX, confocal microscopy studies determined that these nanoparticles are rapidly internalized by the cells.

## 3. Stimuli-Responsive Systems Based in Exogenous Activity

Systems based on exogenous stimuli work like a switch. When activated by an external stimulus to the human body (light, magnetic field, and ultrasound), their physicochemical properties are modified, and the release of the therapeutic agent incorporated in the nanoparticles into the desired tissue or cells is controlled. However, the nanocarrier cannot carry out the release without the presence of this stimulus [44,45]. Table 2 presents some of the most significant proposals for exogenous or external stimuli-responsive systems.

### 3.1. Magnetic-Responsive Systems

The nature of the applied magnetic field is a property used to develop magnetically responsive nanoparticles. While there are nanoparticles that are magnetically guided towards the specific tissue to carry out the drug release due to the presence of a permanent magnetic field, there are other systems that, due to the presence of an alternating magnetic field, experience a temperature increase, which causes the destabilization of the structure and drug release [46]. These types of systems are based on hybrid core–shell nanostructures formed by a paramagnetic or superparamagnetic component and silica. While the magnetic component is the one affected by the external stimulus, the silica provides stability, decreases toxicity, and improves biocompatibility [47]. Moreover, due to the various advantages of silica, including its easy functionalization, different functional groups can be incorporated, allowing interactions with small molecules, directing molecules and therapeutic agents. This results in very versatile systems.

In a recent study, MSN-based magnetically responsive nanoparticles were developed with monodispersed Fe_3_O_4_ nanoparticles as capping agents [48]. Specifically, Fe_3_O_4_ nanoparticles act as plugs that prevent the release of molecules hosted inside the pores. This is possible thanks to the covalent interaction between the 3-aminopropyl groups on the MSN surface and the iron oxide nanoparticles functionalized with meso-2,3-dimercaptosuccinic acid (MSN@Fe_3_O_4_). Subsequently, the CPT was enclosed in the pores. In this way, the iron nanoparticles form a dense and uniform layer tightly bound to the MSN Surface. Therefore, no CPT leakage from the nanoparticle pores occurs as long as there is no magnetic stimulus to detach the iron nanoparticles from the structure. Moreover, it has been observed that the energy induced by the magnetic stimulation for various time intervals is large enough to cause the scission of the covalent bond between the iron nanoparticles and MSNs. Consequently, the rate of removal of the nano-Fe_3_O_4_ caps and, with it, the release and diffusion of the drug can then be a function of the magnetic energy. In vitro assays against human lung adenocarcinoma epithelial cell line A549 showed that CPT-loaded MSN@Fe_3_O_4_ nanoparticles without magnetic stimulus exhibit high cell viability by preventing CPT release from MSN. However, under magnetic stimulation, cell viability decreased to 42%. This effect is probably due to the drug release caused by the detachment of the nanoparticles from the pores.

**Table 2:** Types of MSN-based external stimuli-responsive systems for drug delivery.

Stimulus	Drug Loading	Release System	Release Mechanism	Ref.
Magnetic	Camptothecin	MSNs capped with monodispersed Fe_3_O_4_ nanoparticles through chemical bond	Chemical bond cleavage	[48]
	Doxorubicin	Monodispersed manganese and cobalt doped iron oxide nanoparticles with a silica shell conjugated with the 4,4′-azobis(4-cyanovaleric acid) as a gate-keeper	Cleavage of the gatekeeper	[49]
Light	Fluorescein disodium and Camptothecin	MSNs modified with an optimal molar ratio of spiropyran and perfluorodecyltriethoxysilane	Conformational conversion of spiropyran	[50]
	Camptothecin	Light-activated mesostructured silica (LAMSs) nanoparticles functionalized with azobenzene moieties	Trans-cis photoisomerization of azobenzene	[51]
	Camptothecin	Nanoimpellers functionalized with azobenzene moieties and a two-photon fluorophore F	Trans-cis photoisomerization of azobenzene	[52]
	Camptothecin	Gold nanoclusters with a homogeneous thin monolayer of amorphous silica (Au@SiO_2_)	Diffusion (promoted by local hyperthermia)	[53]
Ultrasound	Topotecan hydrochloride	MSNs functionalized with poly(ethylene glycol) and 4,4′-azobis(4-cyanovaleric acid)	Cleavage of the azo moiety of the thermosensitive linker	[54]
	Gadopentetate dimeglumine Gd(DTPA)^2−^	MSNs with pores capped with poly(ethylene glycol)	Poly(ethylene glycol) bond cleavage	[55]

An innovative system based in a core@shell structure consisting of a manganese- and cobalt-doped iron oxide nanoparticles cores and a mesoporous silica shell (MnFe_2_O_4_@CoFe_2_O_4_@MSN = Mag@MSN) was developed for spatial and temporal control of drug release [49]. Initially, the silica shell was modified with 3-(aminopropyl)-triethoxysilane (APTS) to functionalize the particle surface with amine groups. Then, conjugation of these amine groups to the free carboxylic acid group of the thermolabile gatekeeper 4,4′-azobis(4-cyanovaleric acid) (ACVA) by nucleophilic substitution allowed covalently attaching ACVA to the particles Mag@MSNs-ACVA. Similarly, the amine group of the 1-adamantylamine (AMA) was covalently coupled to Mag@MSNs-ACVA via an amide bond with the free carboxylic acid of ACVA using carbodiimide chemistry (Mag@MSNs-AMA). In the last step, DOX was loaded into the pores of Mag@MSNs-AMA, followed by the introduction of *β*-cyclodextrin (*β*-CD) to bind with the AMA ligand and form a non-covalent supramolecular host–guest complex to block the pores and prevent premature release of DOX. With the application of an alternating magnetic field (AMF), gatekeeper cleavage triggered by the temperature increase causes the C-N bond of the ACVA ligand to fragment. This frees the pores and allows the charge to diffuse through them. In vitro assays against human PANC-1 pancreatic cancer cells confirmed that, while nanoparticles without DOX did not affect viability in the presence of the AMF as an external stimulus, nanoparticles with DOX caused a marked decrease in viability that increased with the duration of the applied AMF. Thus, it was corroborated that the heat generated by exposure to AMF does not cause damage to the cells and can control the amount of charge released by the time of exposure to AMF

### 3.2. Light-Responsive Systems

This field has focused the efforts of many scientists due to the significant advantages of light as an external stimulus in the field of biomedicine. This type of nanoplatform has light-sensitive materials due to the presence of molecules with photoactive groups that change their properties when irradiated with ultraviolet (UV), visible (Vis), and/or near-infrared (NIR) light, resulting in the release of the therapeutic agent. A significant feature of light is that it can be localized in time and space, allowing for greater safety and specificity [56,57]. In addition, other properties that make light advantageous for use as a stimulus in materials development are its non-invasiveness, convenience, and ease of use [58]. However, both ultraviolet and visible light have low tissue penetration and can be detrimental for in vitro and in vivo applications. The near-infrared region (650–900 nm) is being widely explored to overcome these limitations. Moreover, light scattering and absorption are minimized in this wavelength region, allowing deeper penetration into tissues without being detrimental [59].

Different MSNs have also been developed for drug delivery applications, such as camptothecin (CPT), to overcome the limitations of this antitumor drug and control drug release as a function of light changes [50,51,52,53]. One such work developed light-activated mesostructured silica nanoparticles (LAMS) to deliver and release CPT into tumor cells upon light activation [51]. These LAMSs were functionalized with azobenzene moieties within the pores. The arrangement of the azobenzene molecules allowed them to bind at one end to the pore walls, leaving the other end free to undergo photoisomerization. The azobenzene molecules, thanks to their freedom of movement, modulated by light irradiation, trap the drug molecules in the nanopores of the nanoparticles, in this case, of CPT. In vitro studies performed on the PANC-1 human pancreatic cancer cell line and the SW480 human colon cancer cell line showed that the nanoparticles caused cancer cell apoptosis after irradiation with 413 nm light for various excitation times (from 0 to 10 min). However, without irradiation, no damage to the cells occurred. This was due to the movement of the azobenzene molecules that was achieved by irradiation, which allowed the pores to open and thus the diffusion of CPT.

Another representative light-responsive prototype was developed by controlling the surface wetting behavior of hybrid mesoporous silica (MS) nanoparticles [50]. In this case, the SNPs were functionalized with amine groups and fluorinated groups by reaction with the corresponding silane to afford the modified MS (MS-FNH_2_). The carboxylic acid terminated spiropyran molecules were conjugated to the amine groups on the surface of the nanoparticles by an amidation reaction. Spiropyran was chosen as the input molecule because its switchable properties give a different hydrophobicity of the closed and open forms. Hybrid nanoparticles were prepared with varying ratios of spiropyran and fluorinated silane, finding that the proportion of spiropyran to fluorinated silane (0.249:1) protected the nanoparticles from being wetted by water. Here, CPT was loaded into the pores through hydrophobic interaction. In vitro assays on EA.hy926 human somatic hybrid cells and HeLa human cervical carcinoma cells showed a superior cytotoxic effect of these nanoparticles after irradiation with UV light (365 nm) compared to non-irradiated nanoparticles. This effect was probably due to the conformational change in the spiropyran under UV irradiation from a closed to an open state, leading to surface wetting and thus CPT release.

Considering the problems arising from the use of UV/Vis light in the biomedical field, including low tissue penetration and cell damage, nanoimpellers functionalized with a two-photon fluorophore F with a high two photon absorption cross-section were developed; suitable for Forster resonance energy transfer (FRET) to photoizomerize azobenzene moieties in the NIR region [52]. For this purpose, the two-photon fluorophore F and azobenzene molecules were co-condensed in the pores of the nanoimpellers (MAF), and subsequently the CPT was physically trapped in the pores. Notably, the two-photon absorption properties of the fluorophore were maintained in the materials and did not decrease after encapsulation. Moreover, to determine the ability of these nanoparticles to induce cell death in MCF-7 human breast cancer cells, MTT assays were performed two days after the cell cultures had been irradiated for a short time with a focused laser beam (input 3 W, output 900 mW cm^−2^). Although the results showed that both MAF particles and nanoparticles functionalized with two-photon fluorophore F (MF) loaded with CPT produced cell death efficiently, in the case of MAF, this effect is much more pronounced under two-photon excitation (TPE).

**Figure 7:**
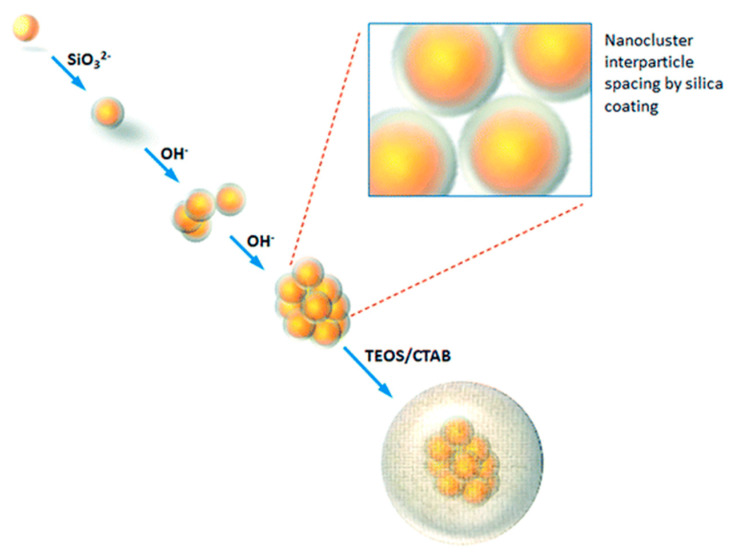
Schematic representation of the synthesis and controlled aggregation of Au@SiO_2_ nanoparticles into gold nanoclusters. (Reprinted with permission from reference [53]. Copyright © 2022 Royal Society of Chemistry, London, UK).

In another study, our group developed a representative light-responsive multifunctional prototype based on plasmonic gold nanoclusters with a protecting shell of porous silica shell (Au@SiO_2_ nanoparticles) [53,60]. The synthesis process of these nanoclusters consists of different stages (Figure 7). Once the gold nanoclusters (GNP) are formed, they are covered with a thin and homogeneous silica layer (1–2 nm). Furthermore, it is worth noting that the aggregation of the GNP in the nanoclusters can be tailored by modifying the alkali conditions. The nanoclusters were the embedded in a thick layer of amorphous silica by the Stöber method to stabilize them under physiological conditions for use in both in vivo and in vitro applications [60]. In contrast, the pores of the hybrid nanospheres were loaded with CPT by an adsorption process. Moreover, this silica wall allowed water vapor diffusion and microbubble formation during the irradiation with NIR light (790 nm), which provoke mechanical disruption of cellular components. Nevertheless, the local heating effect corresponding to the absorption of NIR radiation also contributed to cell death. In vitro study in human 42-MG-BA glioma cells showed that the combination of chemotherapy and photothermal effects enhanced cell death compared to nanoclusters that did not incorporate the drug or were not irradiated. 

### 3.3. Ultrasound-Responsive Systems

In recent years, ultrasound (US) has attracted great interest in the development of systems sensitive to this stimulus due to its non-invasive nature, its ability to penetrate living tissues, and its spatio-temporal control [61]. In this way, it is possible to avoid exposure to surrounding healthy tissues while acting only on tumor tissues, and thus reduce side effects. However, ultrasound waves can cause drug release from an SRS due to the alteration they cause in the structure of the SRS. Two phenomena can cause this modification in the structure of the drug delivery system: (i) thermal and/or mechanical effects associated with cavitation phenomena, (ii) radiation forces. 

In a promising study, a hierarchical US-responsive MSNs based on PEGylated MSNs was developed. MSNs functionalized with amine groups were conjugated to the cleavable thermosensitive linker 4,4′-azobis(4-cyanovaleric acid) (ABCVA) [54]. Subsequently, PEG was covalently attached to this linker by amidation (MSN-NH_2_-T-PEG). Particles without the linker (MSN-NH_2_-PEG) were also prepared for use as a control. With the aim to corroborate whether complete cleavage of the heat-sensitive ABCVA linker occurred, suspensions of different particles were treated at 70 °C and at 37 °C (control). For this assay, PEG was labelled with fluorescein. While fluorescence intensity was detected for MSN-NH_2_-T-PEG at 70 °C, no significant fluorescence was found for the samples without the thermosensitive linker (MSN-NH_2_-PEG) and for the control samples treated at 37 °C. Furthermore, to study the influence of ultrasound on the detachment of PEG chains by the cleavage of the azo linker, the nanoparticles were exposed to ultrasound at a frequency of 1.3 MHz for 20 min, finding that the temperature increased up to 40–45 °C. It was not necessary to reach 70 °C to release the PEG chains. Flow cytometry assays confirmed a significant increase in MSN-NH_2_-T-PEG uptake after US treatment, but no significant differences were observed for MSN-NH_2_-PEG. An in vitro study in HOS cells confirmed that only MSN-NH_2_-T-PEG loaded with topotecan, a camptothecin analogue, resulted in increased cell death after US exposure, demonstrating that the thermosensitive linker and ultrasound enhance cellular uptake.

**Figure 8:**
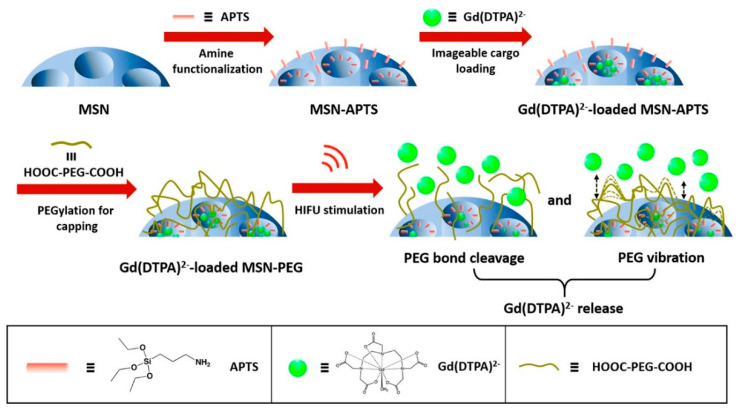
Schematic illustration of the synthesis of new MRgHIFU-responsive platform based on MSNs. (Reprinted with permission from reference [55]. Copyright © 2022, American Chemical Society, Washington, DC, USA).

One of the most interesting models of nanoplatforms that can deliver cargoes by applying high-intensity-focused ultrasound (HIFU) to a previously designated localized spot, which can be monitored and visualized in real-time by NMR, is based on nanoparticles coated with PEG chains [55]. For this purpose, the surface of MSNs was functionalized with 3-aminopropyltrimethoxy silane groups (Figure 8). Subsequently, the FDA-approved gadolinium-based MRI contrast agent, gadopentetate dimeglumine (Gd(DTPA)^2−^), was loaded into the pores. Finally, the pores were capped with PEG through amidation of the amine groups on the surface of the MSNs and the acid group of the PEG chains, preventing the leakage of the MRI contrast agent molecules. 

The most important feature of this model is that drug release occurs solely and exclusively by MRI-guided high-intensity-focused ultrasound (MRgHIFU) activation when MRI tracking of the nanovehicle confirms that it is in the tumor tissue. HIFU simulation tests (74 W, 3 min) showed that the release of the charge occurs by mechanical disruption of the PEG chains and with a temperature increase of no more than 4 °C, which reduces the possibility of causing thermal damage to healthy tissues, becoming one of the significant advantages of this system. The amount of Gd(DTPA)^2−^ released was controlled by the HIFU stimulation times and power levels with this technology. On the other hand, thanks to these nanoparticles, the dose of Gd(DTPA)^2−^ needed to enhance the contrast of the T1-weighted MR image can be reduced to the same level as free Gd(DTPA)^2−^, which means a decrease in toxicity and side effects caused by the accumulation of gadolinium in the body.

## 4. Targeting Molecules

Specific diffusion to cancer cells is mandatory in order to achieve high efficacy in chemotherapy with minimum effects over healthy tissue [62]. Here, it must be considered that when nanoparticles reach the bloodstream, passive targeting to tumor is usually promoted by the partial degradation of endothelium during the angiogenesis process, which allows accumulation into tumor surrounding area (enhanced permeability and retention effect, EPR) [63]. However, once there, only biological recognition may improve the selectivity of the treatment, with specific interactions ligand–receptor driving particle endocytosis into the malign cells [37]. 

Targeting moieties are usually incorporated on nanoparticle surface by covalent linking, mostly ester bond, amide bond and maleimide–thiol coupling [64], and the success of molecular recognition depends on the overexpression of the targeted receptors and, specially, on the affinity between the complementary blocks. According to the molecular size and chemical nature, tumor targeting molecules can be classified into five categories: (i) small molecules; (ii) polysaccharides; (iii) peptides; (iv) aptamers; and (v) antibodies. We, here, present some of the most relevant examples of application in silica-based nanomedicines.

Probably the most used targeting component has been folic acid (FA), based in the overexpression of FOLH1 receptors in many cancer types. For instance, MSNs functionalized with FA and camptothecin (CPT) have been used for targeted therapy of human breast cancer in xenograft mice, showing a slight but not significant increase in the tumor-suppressing effect [65]. These authors explain that this unexpected result could be due to the high CPT dosage and the lack of folate receptor expression in MCF-7 cells. In a further study, these authors developed silica-based nanomedicines using the so-called nanovalve concept, and functionalized with FA. In vitro testing showed active internalization when FA was a part of the nanocarrier surface [66].

Polysaccharides are usually applied as biodegradable coatings to nanomedicines, but some of them have shown targeting properties to specific tumor cells. Hyaluric acid (HA) interacts with overexpressed receptor in cancer cells cluster determinant 44 (CD44) and receptor for HA-mediated motility (RHAMM) [67]. Through interaction with receptors, HA allow enhanced targeting in cancer therapy. Here, HA has been incorporated over doxorubicin-loaded MSNs due to its specific affinity to CD44 receptors, overexpressed on human colon cancer cell line HCT-116. The resulting conjugated showed stronger cytotoxic activity to HCT-116 cell than the free drug and non-targeted nanoparticles, due to the enhanced cell internalization behavior of HA-MSNs [68].

Interestingly, some peptides have exhibited potential for tumor targeting in drug and gene delivery, despite their low specificity for antigens [37]. Recently, J. Binker’s group proposed a modular design of a protocell system, in which a peptide targeting specific cancer cells overexpressed protein receptors (e.g., IL-11Rα, GRP78, and EphA5) is conjugated to a core–shell material, with a lipid bilayer coating, and a silica nucleus containing the therapeutic cargo. Specific tumor therapies were proposed, by exchanging the targeting peptide and/or therapeutic cargo [69]. In addition, SNPs, MSNs, and hollow MSNs have been functionalized with epidermal growth factor receptor (EGFR), an anticancer drug target for a number of cancers, such as non-small cell lung cancer, resistance colorectal cancer, and hepatocellular carcinoma, obtaining cell growth inhibition from 64 to 85% over a range of chemo and genetic therapies [70,71,72,73]. 

Aptamers are single-stranded DNA or RNA with 20–100 nucleotides that specifically bind to antigens forming three-dimensional structures. As protein antibodies, aptamers bind to cell membrane receptors and mediate conjugated nanoparticles to enter into cells. However, aptamers have several advantages over standard antibodies, such as smaller size, high binding affinity and specificity, high stability in physiological medium, good biocompatibility, and low immunogenicity [74]. In this sense, a nanohybrid of mesoporous silica with carbon loaded with doxorubicin and targeted with HB5 aptamer, found a significant improvement in cell uptake and cytotoxic activity over HER2-positive breast cancer cells, by specific interactions with HER2 cell membrane receptor [75]. Moreover, MSNs loaded with emtansine (also named DM1) and surface-decorated with dopamine and a polyethylene glycol, and epithelial cell adhesion molecule (EpCAM) aptamer showed good efficacy against colorectal cancer in xenografts (mice) of the SW480human colorectal cancer cell line, with about 90% tumor volume reduction in two weeks [76]. 

However, the most specific targeting ligands are antibodies. They present outstanding antigen-recognition capacity, and have been used frequently as targeting components in SNPs and MSNs [37,64]. Unfortunately, they are normally very sensitive to physical and chemical conditions, which hinder their covalent bonding over silica surface by standard protocols. Furthermore, they may induce a strong immune response under blood exposure, leading to protein corona formation and removal from plasma by macrophages [77]. To overcome these limitations, particles are usually coated by a protecting shield of PEG. Moreover, it is usual to introduce long cross-linkers to connect the antibody molecule to the silica wall, avoiding possible interferences in chemical coupling by other surface moieties [31,78,79]. 

**Figure 9:**
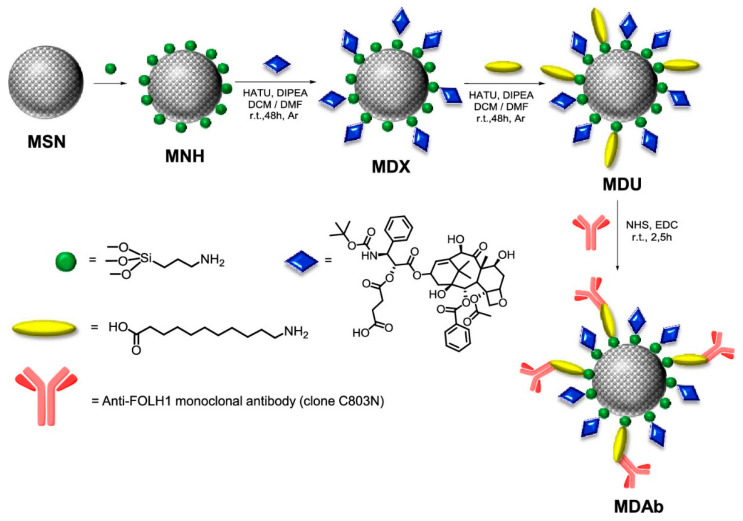
Artistic representation of the multistep synthetic process for preparation of multifunctional MSNs. Legend: MSN: mesoporous silica nanoparticle; MNH: MSN functionalized with amino groups; MDX; MNH functionalized with docetaxel; MDU: MDX functionalized with undecanoic acid (cross-linker); MDAb: MDX functionalized with anti-FOLH1 monoclonal antibody. (Reprinted with permission from reference [31]. Copyright © 2022 American Chemical Society, Washington, DC, USA).

In this context, although many papers have tackled the use of nanoparticle/antibody conjugates for cancer therapy [80,81,82], the first report about targeted therapeutic systems against prostate cancer (PCa) based on SNPs was recently presented by our group (Figure 9). It has been reported that DDS conjugation with anti-FOLH1 (aPSMA) monoclonal antibody provides a substantial improvement in the capacity of these nanovehicles to binding specifically to cells that express PSMA receptors [83], enhancing the cytotoxic effect of the nanoplatforms. In this sense, our data suggest that selectivity and high affinity of MSNs can be attributed to the specific interaction between antibody conjugated nanoparticles loaded with docetaxel and the receptor expressed on the membrane of human prostate adenocarcinoma LNCaP cell line. Therefore, aPSMA antibody incorporation in this docetaxel delivery system may promotes drug diffusion to malignant cells (over 25% of increased cell internalization), minimizing side effects over healthy tissue. This should allow the administration of higher doses of the drug in the case of chemotherapy resistance. This is particularly relevant in this type of cancer, as PSMA expression is correlated with tumor invasiveness but, in the case of intraprostatic administration, the targeting effect can promote local accumulation on malign nodules, improving DTX cytotoxic effect with no significant damage over the surrounding tissue [84].

## 5. Clinical Testing

Despite the myriad of articles and patents already published on the field, currently no silica-based nanomedicine has completed the clinical stage satisfactorily. There are two main issues that preclude medicine agencies (e.g., FDA and EMA), from giving direct approval to silica-based formulations: (i) No long-term in vivo preclinical toxicity studies are available, yet. A one-year chronic toxicity evaluation of intravenously administered non-surface modified SNPs, indicate that female and male BALB/c mice need up to one year to recover from acute tissue toxic effects of SNPs upon single dose intravenous (IV) administration at their 10-day maximum tolerated dose, prompting the need for monitoring carefully particle physico-chemical properties (e.g., size, shape, surface charge and, mostly, and organic coating) in order to minimized toxic effects [85]. (ii) Most of the silica-based DDSs are hybrid materials, taking advantage of functional properties of several inorganic moieties or inorganic and organic components. However, from the regulatory point of view, this is much more challenging, as it requires the evaluation of every single component, which possibly will delay clinical translation [86]. So far, there are some candidates for drug delivery, imaging, and theranostics systems that are currently in Phases I and II, showing the potential of silica nanoparticle-based formulations [87]; we have compiled them all in Table 3. 

Unfortunately, currently no preparation of SNPs with antitumor drugs has obtained approval for the clinical use albeit, according to the milestones already achieved with other silica-based systems, we expect this will happen in the near future. In this context, although SNPs have shown good tolerability in oral administration, including improving the PK of some hydrophobic drugs [88,89,90], the most usual administration route is IV. Here, hybrids of silica with plasmonic nanoparticles (gold nanoparticles and gold nanoshells) have found application for the thermal ablation of tumors that are difficult to fully remove surgically [91], as well as to reduce the risk of coronary atherosclerosis [92]. Furthermore, ultra-small SNPs (Cornell dots) [93] can be applied for clinical imaging by the incorporation of SNPs on fluorescence moieties (e.g., Cy5.5), or positron emission tomography (PET) radiotracers (e.g., ^89^Zr, ^64^Cu, and ^124^I). This allows the detection and localization of the in-tissue malignant nodes of different cancers, also guiding the biopsy with high accuracy and lower risk. 

**Table 3:** Silica-based nanomedicines under clinical investigation ^a^.

Material	Clinical Trial	Patients	Status	Action	Active Agent	Pathology	Via	Outcome	Ref.
Lipoceramic (silica@lipid)	Clinical Study	16	Completed	Bioavailability study	Ibuprofen	---	Oral	Improved PK	[88]
	ACTRN 12618001929291	12	Completed	Bioavailability study	Simvastatin	---		Improved PK	[89]
MSN	Clinical Study	12	Completed	Bioavailability study	Fenofibrate	---	Oral	Improved PK	[90]
Au@SiO_2_ and Au/Fe_3_O_4_@SiO_2_ (core–shell)	NCT01270139	180	Completed	Photothermal therapy	Gold nanoparticles	Atherosclerosis	IV	Reduced coronary atherosclerosis	[92]
	NCT01436123	62	Terminated	Photothermal therapy	Gold nanoparticles	Atherosclerosis	IV	Reduced risk of atherosclerosis	[92]
Aurolase (SiO_2_@Au)	NCT00848042	11	Completed	Photothermal therapy	Gold nanoshells	Head and neck cancer	IV	Tumor ablation	[91]
AuroShell (SiO_2_@Au)	NCT02680535	45	Completed	Photothermal therapy	Gold nanoshells	Neoplasms of the prostate	IV	Pending ^b^	[91]
	NCT04240639	60	Recruiting	Photothermal therapy	Gold nanoshells	Neoplasms of the prostate	IV	Pending ^b^	[91]
Cornell dots (ultra small SNPs)	NCT03465618	10	Recruiting	PET Imaging, Fluorescent Imaging	^89^Zr, Cy5.5	Malignant brain tumors	IV	Pending	[93]
	NCT02106598	86	Recruiting	Fluorescent Imaging	Cy5.5	Melanoma	IV	Pending	[93]
	NCT01266096	10	Active, not recruiting	PET Imaging	^124^I	Melanoma and malignant brain tumors	IV	Pending	[93]
	NCT04167969	10	Recruiting	PET Imaging, Fluorescent Imaging	^64^Cu, Cy5.5	Prostate cancer	IV	Pending	[93]

^a^ NCT trials: Additional information may be found at www.clinicaltrials.gov (accessed on 7 December 2021). ^b^ A former pilot study over 16 patients described in reference [90] showed successful tumor ablation in prostate cancer patients.

## 6. Conclusions and Future Direction

So far, the efficacy of silica nanoparticle formulations for the precise delivery of anticancer drugs, tumor elimination, and relapse inhibition has been already proved in many preclinical studies. However, despite so many silica-based nanomedicines having been proposed, some of them currently at the clinical stage, the main goal still to be accomplished in order to achieve complete development, including the corresponding Medicine Agency approval for clinical trials, industrial production in good manufacturing practices (GMPs), and commercialization, is to ensure the absolute lack of long-term toxicity of these preparations. In this sense, there are different pathways in order to achieve this target, here we give some of the possible alternatives.

(a) Small particle diameter: a very small particle diameter (e.g., <10 nm) can favor particle elimination by renal filtration. This has been conducted in the case of Cornell dots [93], with no significant side effects due to a short plasma half-life (<9 h). This is an interesting property for clinical imaging agents, but is not recommendable for drug delivery systems, as the smaller particles may extravasate before reaching the target cells, reducing the therapeutic response, and leading to severe undesired effects.
(b) High drug loading: the higher the drug content in the nanomedicine, the lower the silica accumulation in tissue. This allows the administered dose to be reduced, and the therapeutic window to be enlarged. Mesoporous materials with well-developed internal geometric structures and high external surface areas for the incorporation of organic groups are probably the best choice for this purpose.
(c) Targeting: as already shown in this review, the incorporation of targeting molecules in the nanoparticles favors tumor accumulation, thus allowing the dose to be reduced.
(d) Organic–silica nanomaterials: hybrid nanomaterials containing silica and organic moieties gathered in a single system basically limit the amount of silica administered. In this review we have presented some potential nanomedicines based on an innocuous and biodegradable organic core (e.g., liposomes, polystyrene, etc.), and a silicate shell containing chemical doors able to be opened by specific stimuli. In these conjugates, the silica content can be reduced as far as 95% with regards the equivalent solid silica nanoparticles.
(e) Replacing silica by structured organic materials. In the last decade, many groups have focused their research on novel materials for bioimaging and drug delivery, as coordination polymers [94,95], and covalent organic frameworks (COFs) [96]. These nanomaterials present well-defined topologies and high surface area, facilitating the incorporation of large quantities of active principles and other functional molecules. Furthermore, they are mostly organic (100% in case of COFs), and can be fully degraded inside the cells releasing their building components, which are later on eliminated by the renal route. In this way, toxicity issues should be no longer an obstacle for the development of novel nanomedicines able to perform efficient and selective chemotherapies, fully free of side effects.
